# Insights into drivers of mobility and cultural dynamics of African hunter–gatherers over the past 120 000 years

**DOI:** 10.1098/rsos.230495

**Published:** 2023-11-01

**Authors:** Johannes Zonker, Cecilia Padilla-Iglesias, Nataša Djurdjevac Conrad

**Affiliations:** ^1^ Zuse Institute Berlin, Berlin, Germany; ^2^ Institute of Anthropology, University of Zurich, Zurich, Switzerland; ^3^ Freie Universität Berlin, Berlin, Germany

**Keywords:** hunter–gatherers, agent-based model, mobility, cultural evolution, spatial model, spatio-temporal clusters

## Abstract

Humans have a unique capacity to innovate, transmit and rely on complex, cumulative culture for survival. While an important body of work has attempted to explore the role of changes in the size and interconnectedness of populations in determining the persistence, diversity and complexity of material culture, results have achieved limited success in explaining the emergence and spatial distribution of cumulative culture over our evolutionary trajectory. Here, we develop a spatio-temporally explicit agent-based model to explore the role of environmentally driven changes in the population dynamics of hunter–gatherer communities in allowing the development, transmission and accumulation of complex culture. By modelling separately demography- and mobility-driven changes in interaction networks, we can assess the extent to which cultural change is driven by different types of population dynamics. We create and validate our model using empirical data from Central Africa spanning 120 000 years. We find that populations would have been able to maintain diverse and elaborate cultural repertoires despite abrupt environmental changes and demographic collapses by preventing isolation through mobility. However, we also reveal that the function of cultural features was also an essential determinant of the effects of environmental or demographic changes on their dynamics. Our work can therefore offer important insights into the role of a foraging lifestyle on the evolution of cumulative culture.

## 1. Introduction

Despite being less genetically diverse than all our Great Ape relatives, humans are able to inhabit every terrestrial habitat of the planet [1]. This unique adaptive ability has been largely explained by our capacity to rely on cumulative culture for survival [2]. Culture is a second inheritance system that parallels and interacts with the genetic system, generating most of human population diversity [3–5]. Cultural variation and innovations accumulate in populations throughout time, allowing for complex cultural adaptations to evolve [6,7].

An important body of theoretical [2,8] and experimental [9,10] work has highlighted the role of changes in the size and interconnectedness of populations in determining the persistence, diversity and spatial scale of material culture [6,10–12]. Moreover, predictions derived from these studies have been increasingly used to try to explain historical patterns of cultural changes and even the appearance of modern human behaviour and cumulative culture [13–16]. For example, it has been proposed that an increase in population interconnectedness may be responsible for the differences between the Upper and the Middle Palaeolithic archaeological records: While the high connectivity of Upper Palaeolithic could have stabilized technological volatility, decreasing the risk of technological losses and increasing demographic robustness, the opposite would have applied to the more fragmented and unstable social networks characterizing the Middle Palaeolithic [16–18]. Nonetheless, other studies have yielded extremely contradictory results [19,20].

For the most part of our evolutionary history, human populations were exclusively composed of hunter–gatherer communities [21]. The mobile nature of these groups is one of the main strategies by which human foragers adapt to changing environments [22–25]. Therefore, changing mobility patterns were almost certainly a key factor influencing the population and cultural dynamics of early members of our species [15,20,26]. However, we are still lacking both formal models and empirical studies that allow an explicit examination of how changing local environmental conditions may interact with social, demographic or geographical factors to determine hunter–gatherer mobility patterns. This has also hindered our ability to predict mobility patterns in the past, and their implications for the emergence and distribution of cumulative culture over our evolutionary trajectory [15,20,26].

In order to overcome these issues, here we develop a spatio-temporally explicit agent-based model (ABM) to examine, on the one hand, the socio-ecological drivers of hunter–gatherer demographic and mobility patterns, and on the other, how changes in such factors over our evolutionary history could have affected the ability of members of our species to invent, exchange and accumulate complex culture. By modelling separately demography- and mobility-driven changes in interaction networks, we can assess the extent to which cultural transitions and patterns of cultural diversity are driven by different types of population dynamics. We then validate our model and test our predictions using empirical data from a real-world setting in Central Africa that homes some of the largest, most resilient and genetically diverged hunter–gatherer populations in the world, with a history of habitation of their current ecological niche dating at least 120 000 years (and possibly much longer) [27–30]. This allows us to explicitly ask how past environmental changes in this region would have altered the demographies of hunter–gatherer populations, their mobility patterns, as well as their ability to interact with one another and subsequently develop, accumulate and exchange different types of cultural traits. Furthermore, by exploring which aspects of hunter–gatherer social structures are required for the emergence and maintenance of complex, cumulative culture we can offer insights into the adaptive nature of a foraging lifestyle.

Concretely, we set to address the following outstanding questions:

1. Theoretical models regarding the effects of demography on cumulative cultural evolution often exclusively focus on traits, such as technology, for which complexity is associated with increased efficiency (and therefore greater pay-offs) [13,14]. In these cases, sequential innovations of greater complexity might accumulate more readily at higher population sizes. It has been argued that when complexity is not associated with greater advantages, as in the case of folk-tales or other stylistic traits, demographic fluctuations might not necessarily affect the cultural repertoire of populations [31,32] (but see also [33]). We therefore model both types of traits (see §2.3) and ask: do demographic fluctuations exert a similar effect on them?
2. The two main factors that have been proposed to mediate the effect of demography on cumulative cultural evolution have been cultural loss and cultural innovation [13,14,34]. If there are too few people, (adaptive) innovations are unlikely to emerge. However, provided that they emerge, they are also more likely to be lost completely by chance. Therefore, we ask: what is the relative role of innovation and cultural loss in determining the diversity and complexity of the cultural repertoires of populations?
3. Cultural evolutionary studies have remarked that the size of the population of agents innovating and exchanging culture does not only depend on its census size, but also on the tendency or ability of its members to interact with one another, i.e. on its social structure [34,35]. Given that hunter–gatherers live in small camps, it has been proposed that their high mobility in the form of frequent changes in camp residence, migration, seasonal gatherings between individuals belonging to different regional groups or even long-range trade or exchange of gifts over hundreds of kilometres [6,36–38] might act as a mechanism for maintaining high levels of cultural complexity and diversity even at low population sizes/densities. Hence, lastly, we ask: could mobility have compensated for demographic collapses during periods of environmental hardship, allowing Central African hunter–gatherers to maintain high levels of cultural complexity and diversity?

## 2. Model formulation

### 2.1. Agent-based model of hunter–gatherer mobility

We formulate our model on the micro-scale as an ABM to resemble approaches described in [39–42]. The basic setting considers a set of *n* agents that follow rules for their spatial movement and social interaction that govern cultural transmission. An agent *α* represents a hunter–gatherer camp that at every point in time *t* has

— *a position* $X_{\alpha }(t)$, representing a location of the camp (agent) in terms of latitude and longitude coordinates within a study area;
— *a camp population* $D_{\alpha }(t)$, representing the number of people who live in the camp (agent);
— *a cultural status* $S_{\alpha }(t)$, representing a cultural tradition or technology of the camp (agent) (see §2.3).

Thus, the state of the agent *α* at time *t* is given by the collection of all three variables and the system state *Y*(*t*) by the collection of the state variables of all agents.

We model the *mobility* of agents as a diffusion process in a landscape, where agents change their position according to the suitability of their physical environment and in reaction to the movements of the other agents. Note that the mobility process does not describe the mobility of the camp members, but the relocation of the camp position, i.e. ‘residential mobility’, which is one of the defining features of hunter–gatherers around the world and has been proposed to have important implications for cultural transmission and evolution [12,22,23,43–45]. The *demographic* changes of the population *D*(*t*) are modelled deterministically with population growth and decline depending on the carrying capacity of the landscape as well as the system state *Y*(*t*). To model *cultural dynamics* and hence track cultural evolutionary processes, we use a common adapted version of Axelrod’s definition [46] in which culture is defined to be a set of attributes that are subject to social influence [34,47]. The culture of an agent (a camp of hunter–gatherers) consists of some number of these attributes, referred to as cultural features, each of which can take a number of values (or traits). This results in agents being monomorphic for each cultural feature. We consider a finite number of *c* features, each represented by an integer value. A feature could be knowledge about a certain kind of tool or technique but also a shared belief, song, story or societal value (see §2.3).

### 2.2. Mathematical modelling of agents’ mobility

In our model, the movement of the agents, i.e. the relocation of camp positions, is governed by the environmental influences, interaction with other agents and possible unknown influences. Formally, these dynamics are generated by the stochastic differential equation (SDE), such that the movement of every agent *α* is given by

2.1 $$dX_{\alpha }(t)=-\nabla (V(X_{\alpha }(t),t)+U_{\alpha }(X(t)))\;dt+\sigma (X_{\alpha }(t))dB_{\alpha }(t),$$

where *V* is the time-dependent suitability landscape of the environment, $U_{\alpha }$ the interaction force between agents, *σ* the friction-dependent scaling function for the noise and $B_{\alpha }(t)$ the standard Brownian motion. A detailed description of all mathematical formulations for the mobility dynamics are given in the electronic supplementary material, §1.2.

#### 2.2.1. Environmental influence

We account for the possible environmental factors by constructing a time-dependent suitability landscape *V* that determines which areas of the domain are attractive for agents [40]. For the construction of the suitability landscape *V*( · , *t*) at time *t*, we use a bio-climatic environmental niche model (ENM) from which we derive the likelihood of a hunter–gatherer camp being present at each position throughout the study area (see [27] for further details on the construction and validation of suitability landscapes. Higher suitability values from the ENM in a particular area correspond to a higher attractiveness of that area for hunter–gatherers (see §3 for details).

#### 2.2.2. Social interaction

The mobility of the agents is also influenced by the positions of other agents, such that agents generate an interaction potential *U* that is similar to physical models for inter-atomic forces [48]. Intuitively, these interaction forces between agents represent the trade-off between avoiding isolation in order to benefit from social interactions for material or cultural exchanges and avoiding conflicts over territories or scarce resources [22,23,25,49]. In our model, interaction forces are constructed such that agents maintain a minimum distance from each other, in order to avoid the overlap of their foraging areas (i.e. areas regularly used for subsistence activities by their members), as has been reported in the literature [12,22,49].

#### 2.2.3. Stochastic effects

The stochastic part of the mobility dynamics is represented by a scaled Brownian motion, and prevents the system from becoming stationary even if every agent has found a position with high suitability and with enough distance from other agents. The scaling of the Brownian motion determines how fast camps can travel, and hence it is dependent on the friction of the terrain. We define a scaling function *σ* such that higher friction of the terrain implies a lower scaling of the Brownian motion. For environmental barriers, e.g. a cliff or a mountain, the scaling function will take values close to 0, such that it is very unlikely that an agent moves through impassable terrain.

### 2.3. Cultural evolution of agents

For changes in the cultural status of agents, we consider two different event types: (i) events that result from intrinsic processes of agents, e.g. the development of a new innovation or loss of knowledge, by *first-order status changes*, and (ii) events that result from exchange or social learning between agents, e.g. the transmission of a trait from one camp to another, by *second-order status changes*. Transmissions between two agents are governed by the agents’ spatial proximity that is captured in the structure of a time-dependent *interaction network*.

Additionally, we consider two types of cultural features that have been modelled in the cultural evolutionary literature: *progressive* and *non-progressive*. The former represent tools whose efficiency and pay-offs increase with an increasing number of component elements, which means that they tend to evolve towards greater complexity [31]. On top of it, these types of tools must be adaptive for the extraction of available resources in specific environments, which may potentially promote high-fidelity copying to prevent ‘maladaptive errors’ and bias the transmission of certain functional (i.e. more elaborate) variants [50,51]. On the other hand, ‘Non-progressive’ cultural features represent those cultural domains not subject to ecological pressures and where complexity is not necessarily associated with greater pay-offs. Examples might be songs, folk-tales, ornaments or other non-technological traits, but the types of cultural dynamics most appropriate for particular cultural features may vary across settings [52–54]. For a mathematically rigorous definition of the cultural evolution of agents, we refer to the electronic supplementary material, §1.3.

#### 2.3.1. Interaction network

We assume that, for the interaction of two agents to occur, spatial proximity is required and construct a network from the agents’ positions *X*(*t*), where two distinct agents *α* and *β* are adjacent at time *t* if their Euclidean distance is closer than a specified interaction radius *r*, i.e. $\|x_{\alpha }(t)-x_{\beta }(t)\|\leq r$. In our model, we assume more frequent interactions, i.e. a higher interaction rate *φ*_1_, in the closer neighbourhood of the agents compared with long-distance interactions that are less frequent with rate *φ*_2_. We thus choose an interaction radius *r*_1_ > 0 for defining short-range and another interaction radius *r*_2_ > *r*_1_ for defining long-range interactions between agents. Based on these assumptions, we define for each time *t* an adjacency matrix *A*(*t*) that represents the time-dependant weighted interaction network between agents, given by

2.2 $$A_{\alpha \beta }(Y(t))\;:=\left\{ \begin{array}{cc} \phi_{1},\; & \text{if}\;\|x_{\alpha }(t)-x_{\beta }(t)\|\leq r_{1}\;\\ \phi_{2},\; & \text{if}\;r_{1}<\|x_{\alpha }(t)-x_{\beta }(t)\|\leq r_{2}\;\\ 0,\; & \text{else. }\; \end{array}\right.$$

**Figure 1.**
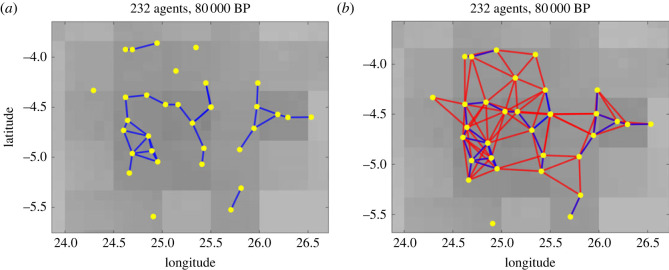
Snapshot of a simulation for illustrating the short-range interactions (*a*) and the weighted interaction network (*b*). Blue edges correspond to edge weights for short-range and red edges to edge weights for long-range interactions.

In figure 1, we plot a part of the interaction network from one simulation at time 80 000 BP.

#### 2.3.2. Progressive cultural features

In the case of progressive features, we consider only gradual changes of trait values, i.e. depending on the type of event, an agent either increases or decreases its trait value by 1. There is no upper bound for possible trait values in order to not artificially limit the possibilities for cultural diversity and complexity. The minimum trait value is 0, which can be interpreted as the lack of knowledge about variants of a particular feature.

*First-order status changes.* We choose constant rates *γ*_*i*_, *λ*_*i*_ > 0 for the events of cultural innovation and cultural loss in each feature *i* and assume no additional dependence on the suitability at the camp position or the trait value itself (with the exception that while an agent is assigned the minimum trait value in a feature the respective rate for cultural loss is set to zero).

*Second-order status changes.* For cultural transmission between agents, we use the interaction network *A*(*t*) to define the rates of each possible event depending on connectivity. We assume that a trait value represents a number of known variants of a cultural feature and thus a difference in trait values can be interpreted as one of the agents being more knowledgeable than the other. Assuming that each variant can be learnt independently, we make the additional assumption that the greater the difference in the trait values between two agents, the more likely it is that an interaction between the two agents will lead to an adoption of knowledge in that feature for the agent with the lower value. This is analogous to people learning from more knowledgeable or skilled individuals [55,56]. An agent can only increase the trait value through interaction if it becomes in contact with an agent that has a higher trait value in that feature. As long as there are neighbours with higher trait values, there is a positive rate for an adoption event leading to an increment in the trait value.

**Figure 2.**
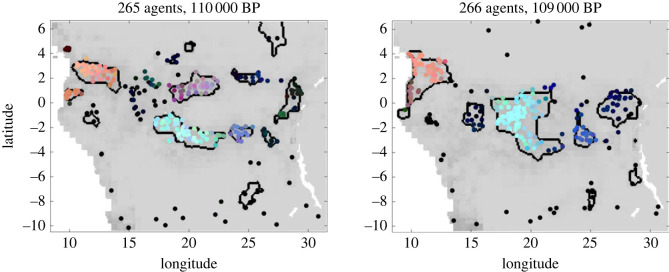
Two consecutive snapshots of a simulation run with *c* = 3 progressive cultural features. Agents are depicted as dots and coloured according to their cultural status. The borders of spatially grouped agents are marked black.

Governed by the rules for the dynamics of progressive cultural features, in figure 2, we plot two consecutive snapshots of one simulation run around 110 000 BP. Here, we consider a case of *c* = 3 features and colour the agents according to their cultural status, such that each trait value denotes one component of the RGB colour vector. Thus, in the resulting plot agents of a similar status are coloured by a similar colour. By construction, brighter colours indicate higher trait values, while darker colours (including black) indicate lower trait values.

#### 2.3.3. Non-progressive cultural features

For non-progressive cultural features, the numerical values of the possible traits are unordered and not associated with a specified complexity. In this case, we define the number of possible trait values as finite and all switches between different trait values are possible.

*First-order status changes.* We choose a constant rate *γ*_*i*_ for spontaneous changes of trait values in each feature that does not depend on the suitability of the agent position or the trait value itself.

*Second-order status changes.* Similar to the progressive case, we assume that interactions between agents require spatial proximity and thus consider the same weighted network defined by the adjacency matrix *A*(*t*). The dynamics of the interaction network, however, differ from those in the progressive case in that agents copy traits of neighbouring agents in a similar fashion to models of opinion dynamics [46]. As long as there are neighbours with a different trait value in some feature, there is a positive rate for a status change event, where an agent copies the trait value of a neighbour.

### 2.4. Demographics

For each agent (i.e. camp), we model growth and decline of its population vector *D*(*t*) as a deterministic process that depends on the local carrying capacity of the environment and the local population. We assume constant rates for exponential population growth and decline, and do not explicitly model microscopic processes within the agents.

The local carrying capacity *K*_*t*_(*x*) is defined for each time *t* as the maximum number of people that can be sustained by foraging within the short-range interaction radius around the location *x* and is assumed to depend linearly on the suitability (see [27] for empirical evidence of this relationship). For each agent, *α* we calculate a local population at the agent position to which the agent *α* itself contributes with its number of camp members. In the case of two agents being close enough to have overlapping foraging areas, they each contribute to the local population of the other agent proportionally to the size of the intersection of their foraging areas. While the local population is smaller than the local carrying capacity, the population of an agent grows exponentially with a constant rate. Otherwise, we assume an exponentially declining agent population.

In reality, the population of a camp cannot grow indefinitely, as after a certain point it can become too large to stay organized as a single unit [57,58]. We thus define a fission threshold *h*_fis_ that sets a maximum for the population size of an agent. If a fission event occurs, i.e. the agent population exceeds the threshold *h*_fis_, the camp represented by agent *α* splits up into two agents with the same position and cultural status as the original agent into which the original agent population is equally distributed.

Given that hunter–gatherer survival and reproductive abilities depend on camp-wide division of labour, cooperation and sharing [43,58–60], we also assume that a camp needs a minimum number of members to be able to survive and thus define a fusion threshold *h*_fus_ that sets a minimum population for an agent [47]. If the population of an agent *α* falls below the fusion threshold *h*_fus_ the members of agent *α* try to get taken in by another nearby agent within their long-range interaction radius. In the case that there is another agent *β* within range, the two agents can merge, which is realized by modifying the agent *β* so that the camp population is increased accordingly and its cultural status adjusted *β* in the progressive case. We assume that knowledge about the variants of a feature is conserved in the merging process which means that the trait value of agent *β* after merging is increased to the trait value of the agent *α* in the case that agent *α* had a higher trait value in that feature, i.e. the merged camp instantly learns all variants known to the camp it merges with. When there are no nearby camps for a fusion process, then camp *α* goes extinct. The number of camps is thus varying in time but ultimately bounded by the carrying capacity of the landscape.

## 3. Application of the model to Central African hunter–gatherer populations

### 3.1. Spatial setting of the model and suitability landscapes

Although the model and analytical tools presented above are designed to be generalizable across temporal, environmental and ethnographic settings, in this section, we parametrize our model with data from contemporary hunter–gatherer populations living in Central Africa. Our suitability landscapes were derived from an ENM built using *N* = 749 contemporary hunter–gatherer camps from Central Africa. Suitability values define the likelihood of a map cell being occupied by contemporary hunter–gatherers, as well as its carrying capacity (*K*_*t*_) [27]. We then used projections of our ENM predictions into 1000- or 2000-year time slices from the present up to 120 000 years before present (BP) using a bias-corrected time series of global terrestrial climate and vegetation to extrapolate our model into the past, and in doing so, obtain suitability landscapes since the last interglacial [27,61]. This time period is of vital importance for our species evolution in Africa as it is when we observe well-defined regional cultural variation across the continent in lithic reduction tools (i.e progressive cultural features) as well as the first evidence for body decoration and hence clear symbolic cultural marking (i.e. cultural diversity in ‘non-progressive’ cultural features) [62,63]. The aim of the present study is not to make conclusive claims regarding the precise cultural dynamics that would have taken place in Central Africa throughout evolutionary history; instead, to demonstrate the potential of our model for testing and discussing cultural evolutionary hypotheses in a spatio-temporally explicit setting of great importance for understanding human history. Future studies should test the results (and parameter settings) from our model against empirical data, to determine the precise drivers of the cultural dynamics that would have taken place in the area over time.

The fact that the resolution of our palaeoclimatic reconstructions ranges from 1000 to 2000 years results in our model having longer phases in which the suitability landscape is stable and some discrete points in time with instantaneous changes in the landscape. In the stable phases of the suitability landscape, the agent distribution can converge temporarily to an equilibrium state, which possibly is disrupted at the time points of change. These piecewise stationary dynamics can only arise if the convergence to a new equilibrium happens on a time scale that is fast enough compared with the frequency of landscape changes. As the accurate modelling of the residential movements and social interactions requires a time step size of one simulated month per time step but resolution of the suitability data is much coarser we update the suitability landscape not in every time step during stable phases and only at the times of landscape changes.

### 3.2. Model initialization and parameter specification

To calculate the potential environmental carrying capacity at each time step, following [27,49] we considered a short-range interaction radius for foraging (*r*_1_) of 20 km of land around each agent (i.e. camp). This radius was determined on the basis of the estimation by Olivero *et al.* [49] of the mean radius (18.5 ± 1.0) encircling a camp that is regularly used for subsistence activities as well the average travel distance covered for foraging activities from 36 studies (21.0 ± 3.65 km).

Fission and fusion thresholds were derived from data on the size of 75 contemporary Central-African hunter–gatherer (CAHG) camps from [27] alongside additional data compiled from the literature and available in electronic supplementary material, dataset 1. These ranged from 8 to 174 individuals (mean=50 individuals; median=47 individuals). After performing sensitivity analyses (electronic supplementary material, §7), we found that a fusion threshold of *N* = 18 was optimum in order to maximize cultural diversity (electronic supplementary material, figures S8 and S9). We also found that diversity in progressive traits (but not in non-progressive traits) increased with increasing fission thresholds up to 60 individuals (electronic supplementary material, figures S4 and S5). Similarly, we found a steep negative relationship between both our fusion and fission thresholds and the ability of agents to accumulate complex traits (mean trait complexity across camps) (electronic supplementary material, figures S7 and S10). This is because higher fusion thresholds result in less populated agents, which in turn may make agents more vulnerable to the loss of adaptive (more complex) variants [14]. At the same time, higher fission thresholds result in less agents in the landscape, and therefore, reduced chances of having models from whom to learn adaptive variants. However, a fusion threshold of *N* = 18 and a fission threshold of *N* = 60 marked an inflection point in this negative relationship, and resulted in a distribution of camp populations that closely resembled that observed in the compiled dataset from contemporary hunter–gatherer populations, with camp sizes with less than 18 individuals or more than 60 individuals corresponding to the smallest and largest 12% of camps (electronic supplementary material, figures S2 and S3).

We then ran 10 model runs, with *c* = 3 cultural features for each combination of parameters on 1 for the 120 000 years for which we had available suitability landscapes. In addition, we recorded every time an agent (camp) moved, and the distance it travelled. Since agents in our model updated their position in each time step we defined agent movements as travel distances that were at least 2 km long. As the computational effort for simulating the ABM is very high, but the results from the mobility model are very consistent, we limited the number of simulations for each parameter setting to a rather small number to enable the exploration of more different settings (see table 1 for the range of the selected parameter values, the electronic supplementary material, table S1 also contains the technical scaling parameters). However, because of this limitation, we restrict the analysis of the cultural dynamics also to macroscopic variables that are behaving consistently throughout the different simulations and the mesoscopic patterns of single simulations.

**Table 1.** Overview of the parameter values used.

parameter	value
foraging radius *r*_*f*_	20 km
starting number of agents *N*_0_	300
fusion threshold *h*_fus_	18
fission threshold *h*_fis_	60
innovation rate (progressive) *γ*_*i*_	0.00001–0.0001
trait value change rate (non-progressive) *γ*_*i*_	0.0001–0.001
loss rate (progressive) *λ*_*i*_	0.01–0.03
population decline rate *ρ*_*d*_	0.001
population growth rate *ρ*_*g*_	0.001
adoption rate (short-range) *φ*_1_	0.02–0.1
adoption rate (long-range) *φ*_2_	0.002–0.01
interaction radius (short-range) *r*_1_	20 km
interaction radius (long-range) *r*_2_	50 km
*σ* (random force scaling)	0.4
Δ*t* (default time-step size)	one month

The agent system was initialized with random positions drawn according to the equilibrium distribution of the initial suitability landscape. The cultural status was initialized at random in the non-progressive case and with the minimum trait value in each feature in the progressive case. We then let the system run for an additional time period, such that the system state of the initial time of our case study already features some level of cultural diversity and also cultural similarity between agents that form a strongly connected interaction network. The numerical simulation was performed using a combination of the Gillespie algorithm and the Euler–Maruyama scheme, which is also used in [40].

### 3.3. Model validation with ethnographic data

Given that our simulation time steps represented one month, we first extracted the number of movements per year, average distance (per move) and total distance moved per year from our simulation and compared them with published data from the Aka and Mbuti Western and Eastern CAHG (respectively) from [58]. We found that our model reproduced very closely the mobility patterns observed in both populations (table 2). This suggests that the modelled relationships between changing suitability landscapes, demography and mobility are representative of those driving the population dynamics of hunter–gatherers currently living in the area (see also electronic supplementary material, dataset 1 for a more extended comparison with the existing literature).

**Table 2.** Mobility of CAHG compared with mobility in our model.

group	res moves/yr	avg dist per move (km)	total distance/yr (km)
Aka	8	7	60
Mbuti	5 to 11	5 to 8	57
Model	9.2	6.2	56.1

**Figure 3.**
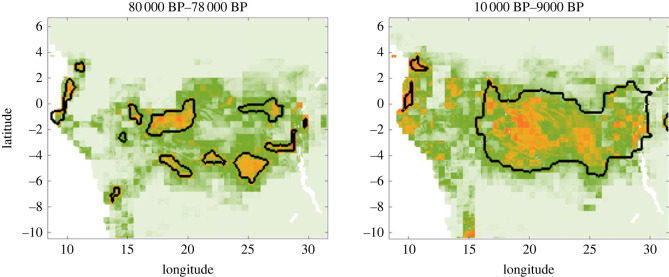
Two snapshots of the time-dependent suitability landscapes *V* at 80 000BP and 10 000BP with borders of mobility clusters marked in black.

Consequently, after verifying that our model accurately reproduced the population dynamics of Central African hunter–gatherers, we could use its predictions alongside the reconstructed past demographies and environmental carrying capacities, to determine the location of clusters of interacting agents at every time period. In doing so, we could assess how changing suitability landscapes would have affected the ability of hunter–gatherer groups to interact with one another. More precisely, we studied how environmentally driven changes in demography and resulting changes in mobility patterns would impact the ‘effective size’ of the populations of agents regularly interchanging culture (or genes) over time [11]. To identify clusters of regularly interacting agents at every time period (henceforth ‘mobility clusters’), we considered the positions of all agents within a time horizon [*t*_1_, *t*_2_] that corresponded to a chosen suitability landscape. Then, we used a hierarchical density-based clustering approach [64] to pinpoint the areas in which the agents were densely connected. As expected, we observe that more fragmented suitability landscapes would have resulted in a greater number of mobility clusters, and therefore, in a reduced region-wide connectivity (figure 3; electronic supplementary material, figures S13 and S14).

## 4. Results

### 4.1. Hunter–gatherer mobility allows the maintenance of cultural differentiation

After identifying mobility-based clusters, we then assessed how these would have impacted cultural dynamics. In doing so, we could gain insights into how environmentally driven changes in regional connectivity could have affected the number of agents potentially exchanging culture, and consequently cultural differentiation over time. For this analysis, we introduced a novel spatio-temporal clustering method to identify culturally connected regions. We defined agents to be close in status if they either had the exact same status in the non-progressive case, or if the Euclidean distance between their status vectors was small in the progressive case (see electronic supplementary material, §5 for more details). We used these definitions of status closeness to construct a time-averaged connectivity matrix *C*([*t*_1_, *t*_2_]) from which we could derive connected components corresponding to clusters based on the cultural status of agents. As time intervals for the matrix construction, we chose the time intervals during which the suitability landscape is static. Via the mobility data we could make the connection between the cultural clusters and the areas of the landscape that the corresponding agents inhabited. We did this by assigning to each location the cultural cluster that most frequently occupied it.

**Figure 4.**
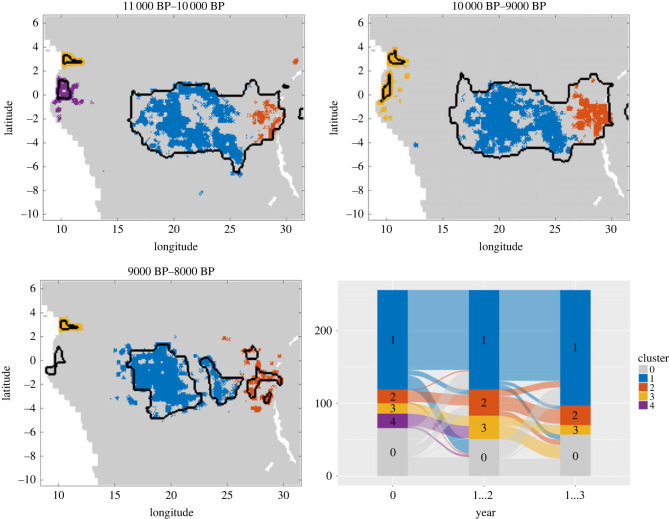
Comparison between clusters based on non-progressive cultural status (coloured areas) and clusters based only on mobility trajectory data (marked by black borders) for three consecutive suitability landscapes centred around 10 000 BP. The relations between the different clusters are illustrated in an alluvial diagram. The simulation parameters are *ϕ*_1_ = 0.08 and *ϕ*_2_ = 0.008 as rates for short- and long-range adoptions, an innovation rate of *γ*_*i*_ = 0.001 and a loss rate of *λ*_*i*_ = 0.02 for each feature *i*.

**Figure 5.**
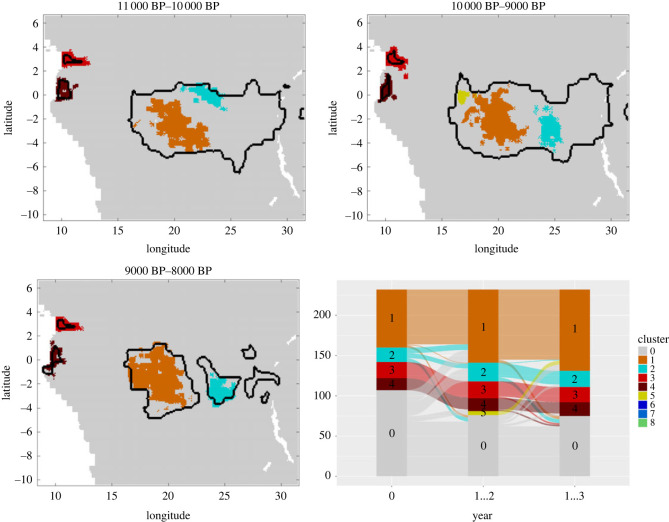
Comparison between clusters based on progressive cultural status (coloured areas) and clusters based only on mobility trajectory data (marked by black borders) for three consecutive suitability landscapes centred around 10 000 BP. The relations between the different clusters are illustrated in an alluvial diagram. The simulation parameters are *φ*_1_ = 0.08 and *φ*_2_ = 0.008 as rates for short- and long-range adoptions, an innovation rate of *γ*_*i*_ = 0.0001 and a loss rate of *λ*_*i*_ = 0.02 for each feature *i*.

Although most of the time agents that were close in space tended to also be close in cultural status, overall, we find that for both types of cultural traits, mobility clusters could encompass several cultural clusters (figures 4, 5 and additional visualizations in the code and data repository). These results imply that cultural differentiation can be maintained even among hunter–gatherer groups regularly interacting with one another, consistent with empirical work showing that contemporary and ancient hunter–gatherer populations tended to be embedded in extremely large social networks encompassing individuals belonging to different camps, bands and even ethnolinguistic units; moreover, that such social organization far from compromised their ability to remain culturally distinct [6,27,36,65–67].

Nonetheless, we also observe that for a given suitability landscape, clusters based on progressive cultural features tend to be smaller and more localized than those based on non-progressive traits (electronic supplementary material, figures S13 and S14). In the absence of differential preferences in terms of the spatial scale of social learning for different types of traits, the fact that in progressive traits, there are some variants that are favoured over others due to their increased efficiency may lead to a more limited diffusion of features across the landscape. In reality, however, we might expect individuals to condition their propensity to acquire these traits on inter-individual distance or cluster membership given that toolkits might be adaptive only in particular ecologies. This could further reinforce the localized nature of subsistence toolkit repertoires, and once more highlights the importance of considering the particular function of cultural elements when trying to assess the impact of ecological, social or demographic features on their dynamics. Taking such properties into account, these visualizations can be compared with maps showing the distribution of actual cultural traditions in the past and present, to gain understanding of the factors driving the spread or disappearance of particular cultural traditions.

### 4.2. The type of cultural features determines the effect of environmental changes on their diversity

**Figure 6.**
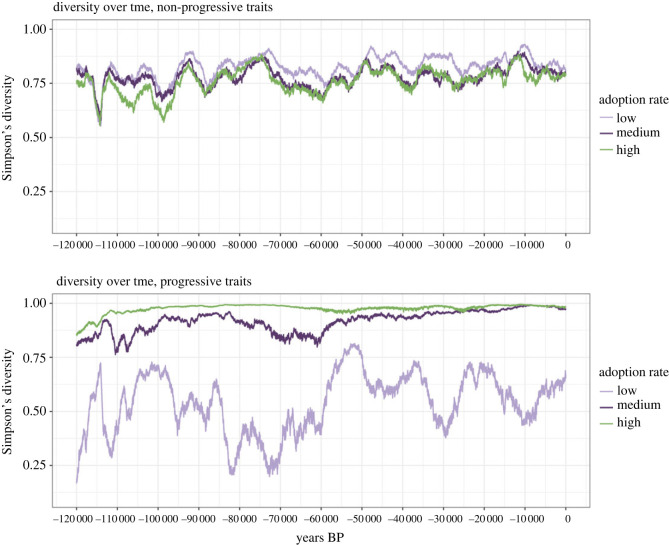
Changes in cultural diversity over time at high innovation rates *γ*_*i*_ and variable adoption rates *φ*_1_, *φ*_2_ and a loss rate of *λ*_*i*_ = 0.02 for each feature *i*. The figure shows the average values over 10 simulations.

When running our model, at each time step we calculated the Simpson’s diversity index for progressive and non-progressive (or opinion) cultural features (electronic supplementary material, §8.4). Simpson’s diversity was calculated, for each type of cultural feature as probability that two agents randomly selected would share the same trait values (for progressive features) or traits (for non-progressive features) across all features. For progressive cultural features, we also calculated the mean trait value across agents in the simulation for each trait (analogous to cultural complexity). When assessing changes in overall cultural diversity throughout our period of study, we observed that agents would have been able to maintain relatively high levels of cultural diversity (figure 6) at all times unless innovation rates *γ*_*i*_ were set to be extremely low (electronic supplementary material, figure S15). Nonetheless, in line with what has been reported in theoretical studies [34,66], we see punctuated increases and decreases in cultural diversity over the time period studied.

On the other hand, for cultural traits for which some variants are more adaptive than others (i.e. progressive traits), and individuals who acquire rare complex variants that can innovate on top of them, a greater effective number of agents may lead to a greater chance of the sequential innovation of such complex variants and a reduction of the likelihood of them getting lost completely by chance [13,14]. However, the effective population size, i.e. the number of agents exchanging cultural information [68] is not only dependent on the number of agents comprising a population but also on the interaction patterns between its members (within-group connectivity) as well as their ability to interact with members of other groups [6,10,16,66,68]. In line with these results, we observed that while for non-progressive traits, the rate of innovation was the main determinant of cultural diversity (figure 6; electronic supplementary material, figure S15), the adoption rates (*φ*_1_, *φ*_2_), i.e. the ability of individuals to socially acquire cultural traits from others, are the main determinants of fluctuations in cultural diversity for progressive traits, as they directly affect the effective population size of agents (electronic supplementary material, table S3). In other words, our results confirm hypotheses that hunter–gatherer inter-camp interactions might be key for preventing the loss of adaptive cultural variants, being able to compensate for low population densities [6].

### 4.3. Social interactions enable the development and maintenance of complex culture even at low population densities

If indeed a larger effective population size of agents helps reducing the probability of losing rare adaptive cultural variants, we would expect a positive relationship between both the number of agents as well as the tendency of agents to exchange culture with one another (i.e. long-range adoption rate) on the mean trait complexity achieved by populations for progressive cultural traits. Indeed, our simulations revealed positive correlations between the number of agents at any given time and the average trait complexity achieved across camps (Pearson’s *r* = 0.265, *p* < 0.001; *r* = 0.426, *p* < 0.001; and *r* = 0.466, *p* < 0.001 at low, medium and high adoption rates, respectively, at high innovation rates and loss rate of 0.02; electronic supplementary material, table S2).

**Figure 7.**
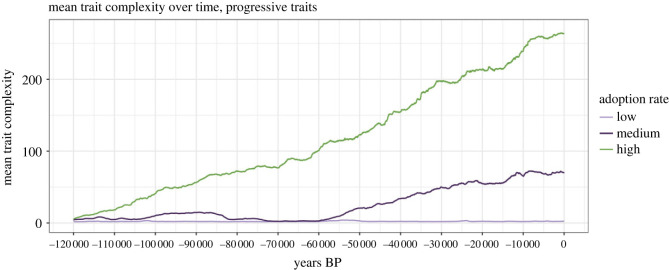
Changes in mean trait complexity over time, at high innovation rates *γ*_*i*_ and a loss rate of *λ*_*i*_ = 0.02 for each feature *i*.

Similarly, we observed that although changing suitability landscapes had a strong effect on the mean trait values over time, as long as the rate of cultural loss (*λ*_*i*_) was smaller than or equal to the long-range adoption rate (*ϕ*_2_), CAHG would have been able to prevent the loss of adaptive cultural variants, even during periods of low environmental suitability (figure 7). In other words, while the reduction in population sizes and connectivity caused by deteriorating environmental conditions would have stagnated the cultural evolution, hunter–gatherers would have prevented the loss of adaptive variants through maintaining a baseline level of interactions with members of other camps. This is also exemplified by the fact that the correlation between population size and mean trait complexity was lower when long-range adoption rates was higher (electronic supplementary material, table S2). Similarly, our simulations illustrate a clear pathway through which isolation can lead to loss of complex skills, and hence to the complete disappearance of some technologies, as has been argued to have been the case in Tasmania following its isolation from continental populations at the start of the Holocene [14]. Our results therefore highlight the importance of connectivity for the ability of cultural traits to increase in complexity, and thus in their adaptiveness over time.

## 5. Discussion

We have presented an ABM aimed at assessing ecological drivers of hunter–gatherer demographic and mobility patterns as well as the impact of such mobility patterns on cultural exchange and evolution over space and time. Furthermore, using data from hunter–gatherer populations in Central Africa throughout the past 120 000 years, we have illustrated the potential of the model to test precise hypotheses in a real-world context over evolutionary time.

Our results add support to a key adaptive role of hunter–gatherer mobility patterns for maintaining the necessary inter-camp connectivity to sustain highly diverse and elaborate cultural repertoires [6,12,68]. The method we develop to visualize clusters of agents based on mobility and of cultural status reveals that extremely high levels of cultural diversity and subpopulation differentiation can be maintained even among highly interconnected populations. Moreover, we found that during periods of reduced population sizes due to deteriorating environmental conditions, hunter–gatherers could have prevented the loss of adaptive variants through maintaining a baseline level of interactions with members of other camps. This was exemplified by the fact that the correlation between population size and mean trait complexity was lower when the rates of sociality (i.e. adoption) were higher. Although, in the present model, mobility strategies were independent of culture, studies have shown that hunter–gatherer groups around the world adapt their mobility regimes to maintain regular contact with members of their cultural group following environmental changes [25,65,69]. Hence, future research including a feedback loop between cultural status and the mobility of agents, such that agents’ cultural status also influences their movement, would shed further light on the implications of the flexible social organization that characterizes hunter–gatherer societies for cumulative culture.

In addition, our findings highlight the importance of considering the function of cultural traits in society when assessing the drivers of their dynamics. For example, while for non-progressive (i.e. symbolic, or non-technological) traits, the rate of innovation was the main determinant of cultural diversity, adoption rates, that is, the ability of individuals to socially acquire cultural traits from others, were the main determinants of fluctuations in cultural diversity for progressive (i.e. technological) traits. In other words, our results confirm hypotheses that hunter–gatherer inter-camp interactions might be key for preventing the loss of adaptive cultural variants, being able to compensate for low population densities. Although in the present example, we implemented these functional differences by including cultural selection for progressive trait values (although not for non-progressive ones), we did not incorporate natural selection in either case. In other words, agents’ trait values did not affect their survival probability or growth rate. Given that cultural differences in some domains can result in differences in the adaptive potential of individuals and groups to particular ecologies (e.g. clothing material in cold environments, traditions of food processing that eliminate naturally present toxins, marriage rules minimizing endogamy etc.) future studies could also consider the effect of natural selection acting on cultural traits [70,71].

Last, the framework we have presented allows us to pinpoint the location of groups of agents regularly interacting and the spatial extent of cultural traditions over time. The flexible nature of our model means that other researchers can adjust the inputs and parametrization according to their area and time scale of study. In turn, these can be compared with and tested against archaeological and ethnographic data to identify patterns and processes promoting changes in population and cultural dynamics such as the spread or disappearance of particular cultural traditions in specific settings.

## Data Availability

The model code is available at https://git.zib.de/bzfzonke/huntergatherermodelpublic. Data used to parametrize the model comes from [27] or is included as electronic supplementary material [72].
